# An Intervention for Sensory Difficulties in Children with Autism: A Randomized Trial

**DOI:** 10.1007/s10803-013-1983-8

**Published:** 2013-11-10

**Authors:** Roseann C. Schaaf, Teal Benevides, Zoe Mailloux, Patricia Faller, Joanne Hunt, Elke van Hooydonk, Regina Freeman, Benjamin Leiby, Jocelyn Sendecki, Donna Kelly

**Affiliations:** 1 Department of Occupational Therapy, Faculty of the Farber Institute of Neuroscience, Thomas Jefferson University, 901 Walnut Street, Suite 605, Philadelphia, PA 19107 USA; 2 Department of Occupational Therapy, Jefferson School of Health Professions, 901 Walnut Street, Philadelphia, PA 19107 USA; 3 Pediatric Therapy Network, Torrance, CA USA; 4 Children’s Specialized Hospital, 200 Somerset Street, New Brunswick, NJ USA; 5 Division of Biostatistics, Thomas Jefferson University, 1015 Chestnut St., Suite M100, Philadelphia, PA 19107 USA; 6 Children’s Specialized Hospital, 94 Stevens Rd, Toms River, NJ 08755 USA; 7 Children’s Specialized Hospital, 150 New Providence Road, Mountainside, NJ 07092 USA

**Keywords:** Autism spectrum disorders, Intervention, Sensory functions

## Abstract

This study evaluated a manualized intervention for sensory difficulties for children with autism, ages 4–8 years, using a randomized trial design. Diagnosis of autism was confirmed using gold standard measures. Results show that the children in the treatment group (n = 17) who received 30 sessions of the occupational therapy intervention scored significantly higher (*p* = 0.003, d = 1.2) on Goal Attainment Scales (primary outcome), and also scored significantly better on measures of caregiver assistance in self-care (*p* = 0.008 d = 0.9) and socialization (*p* = 0.04, d = 0.7) than the Usual Care control group (n = 15). The study shows high rigor in its measurement of treatment fidelity and use of a manualized protocol, and provides support for the use of this intervention for children with autism. Findings are discussed in terms of their implications for practice and future research.

## Introduction

Difficulty processing, integrating and responding to sensory stimuli has been described as a feature of autism spectrum disorders (ASD) since the disorder was first identified. Current estimates show that between 45 and 96 % of children with ASD demonstrate these sensory difficulties (Ben-Sasson et al. 2009; Lane et al. 2010) and sensory features (i.e.: hyper- or hypo reactivity to sensory input or unusual interest in the sensory aspects of the environment) are now included as one of four possible manifestations of ‘Restricted, Repetitive Patterns of Behavior, Interests, or Activities’ (American Psychiatric Association 2013). Families report that behaviors associated with difficulty processing and integrating sensory information create social isolation for them and their child, restrict participation in daily living activities (Schaaf et al. 2011) and impact social engagement (Hilton et al. 2007, 2010; Baker et al. 2008; Ashburner et al. 2008; Reynolds et al. 2011; Watson et al. 2011; Hochhauser and Engel-Yeger 2010). Consequently, interventions to address problems associated with difficulty processing sensory information, such as occupational therapy using sensory integration (OT/SI), (Ayres 1972, 1979, 1989) are among the most often requested services by parents of children with ASD (Mandell et al. 2005; Green et al. 2006; Goin-Kochel et al. 2009). There is emerging evidence regarding positive outcomes of OT/SI for children with ASD (Pfeiffer et al. 2011; Fazlioglu and Baran 2008; and see Schaaf 2011 for a review), however, methodological limitations preclude definitive conclusions. Hence, there is the need for a rigorous study of OT/SI that includes a manualized protocol and measurement of treatment adherence (Case-Smith and Arbesman 2008; Watling et al. 2011). Fortunately, a validated measure of treatment fidelity that describes the key principles of the sensory integrative approach and provides guidelines for best practice is now available (Parham et al. 2011, 2007; May-Benson et al., in press). Importantly, this measure provides a means to evaluate the fidelity of OT/SI in a clinical trial while assuring internal and external validity; a standard that is followed in the current study.

A second advancement that enhances the testing of this intervention is data showing that Goal Attainment Scaling (GAS) is a useful outcome measure for studies of interventions for ASD (Ruble et al. 2012). GAS is used to measure functional and meaningful aspects of an individual’s progress (Mailloux et al. 2007; Kiresuk et al. 1994). In autism, inherent heterogeneity often confounds findings, and thus, it is important to utilize outcome measures that are sensitive to individual outcomes. GAS has been shown to be a substantive and sensitive approach to evaluate progress on individualized goals in randomized controlled trials of psychosocial interventions for children with autism provided that specific quality indicators are present. These include that goals are independently rated, evaluated for equivalence between groups (comparability), scaled with equidistance, have measurable criteria, and clear, identifiable benchmarks (Ruble et al. 2012), recommendations that we followed in this study. A further strength of using GAS is that it provides a means to identify and measure outcomes that are parent-chosen and thus, meaningful to family. Given the increased emphasis on measurement of outcomes that are meaningful to the client or family (PCORI, http://www.pcori.org), the use of GAS provides a model for best practice.

Given the need for a rigorous randomized trial of OT/SI for individuals with ASD, the primary purpose of this study is to evaluate the efficacy of OT/SI following a manualized protocol on individual goal attainment (primary outcome) in comparison to usual care (UC). The secondary purpose was to evaluate the impact of this approach on the child’s sensory behaviors, adaptive behaviors and functional skills.

## Methods

### Participants

Thirty-two children participated in this study. A convenience sample of eligible families was recruited from the children’s hospital where the study took place and the surrounding community. Families were eligible to participate if their child: (1) was between the ages of 4.0 and 7.11 at the time of enrollment, (2) had a diagnosis of an autism spectrum disorder from a licensed psychologist based on the results of the Autism Diagnostic Interview-Revised (ADI-R) (Lord et al. 1994) and the Autism Diagnostic Observation Schedule (ADOS) (Lord et al. 1999), (3) had a non-verbal cognitive level of >65 (this IQ cut score is based on findings from an earlier study where we assessed the feasibility of conducting this intervention with children with ASD—Schaaf Benevides et al. 2012); (4) demonstrated difficulty processing and integrating sensory information as measured by the Sensory Profile (SP—Dunn 1999; 3 or more subscales or total test score in the definite difference range) or the Sensory Integration and Praxis Test (SIPT- Ayres 1989; score of<−1.0 on 3 or more subtests); and (5) parents were willing to attend 3 weekly sessions for the duration of the 10-week study period and to refrain from initiation of any new treatments including medications during the study period.

Child characteristics are also shown in Table 1 below for the treatment (*n* = 17) and UC control group (*n* = 15). In keeping with current gender prevalence estimates of ASD (CDC, 2009), the majority of the participants in both groups were boys (Treatment: 14 males, 3 females; UC: 12 males, 3 females) and Caucasian (treatment: 16 White, 1 not-reported; UC: 13 White, 2 Asian). Highest parent-reported level of education in both groups was similar, with 11 (65 %) parents in the treatment group reporting a 4-year college degree or higher, and nine (60 %) parents in the UC group reporting a 4-year degree or higher. Age, autism severity, cognitive level, and non-study related services were similar between the two groups. Non-project services, or “usual care” (UC) received during the study period was similar between the groups and documented by parents logging their child’s weekly services in hours per week. Usual care included non-study related services such as speech and language services, behavioral interventions, educational program and other therapies as described in Table 1.

**Table 1:** Child characteristics and non-study services received

	OT/SI *n* = 17	Usual care *n* = 15	*p*
Age (mos)			
Mean (SD)	71.35 (14.90)	72.33 (10.81)	*t*(30) = 0.21, *p* = 0.84
Range	56–86	62–83	
Full scale IQ			
Mean (SD)	89.75 (18.74)	91.86 (11.93)	*t*(28) = 0.36, *p* = 0.72
Range	59–123	64–109	
Non-verbal IQ^a^			
Mean (SD)	91.87 (17.48)	95.00 (10.03)	*t*(28) = 0.60, *p* = 0.55
Range	55–119	31–79	
Verbal IQ			
Mean (SD)	93.56 (18.33)	93.79 (14.26)	*t*(28) = 0.04, *p* = 0.97
Range	63–135	69–114	
ADOS autism severity score			
Mean (SD)	7.76 (1.6)	8.40 (1.6)	*t*(30) = 1.09, *p* = 0.28
Range	5–10	6–10	
*Other services* ^a^			
Total behavioral treatments (ABA home, ABA school, in hours)			
Mean (SD)	8.94 (19.38)	23.3 (63.00)	U = 112.0, *p* = 0.77
Median	0	0	
Range (hours)	0–72	0–240^b^	
Frequency of children receiving	5	3	
Occupational therapy, school (hours)			
Mean (SD)	10.95 (14.81)	10.78 (9.29)	U = 110.0, *p* = 0.50
Median	8	10	
Range	0–45	0–32	
Frequency of children receiving	8	12	
Pharmacological treatments (*f)*			
Not on medications	13	12	FET, *p* = 0.99
On medications	4	3	
Clonadine	1	0	
Antidepressant	0	3	
Methylphenidate	2	1	
Adderal	1	0	

*FET* fisher exact test

^a^One participant randomized to treatment had a combined IQ of 65 (non-verbal IQ = 55 and a verbal IQ = 77)

^b^One participant in the control group reported receiving 240 h of behavioral support in school

### Overview and Timeline

**Fig. 1:**
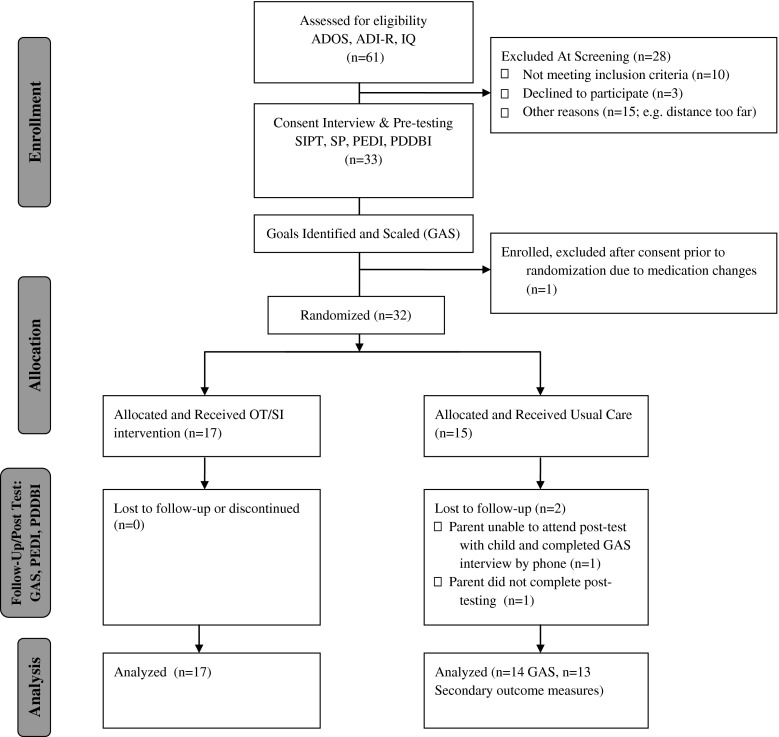
Participant recruitment, enrollment, randomization and retention

Data for this randomized clinical trial were collected at a single project site in central New Jersey, between 2010 and 2012. The study was approved by the first author’s research ethics committee. Figure 1 provides an overview of the recruitment, enrollment, randomization and retention flow. Following phone screening for eligibility with interested parents, child participants were scheduled for confirmation of autism diagnosis using the ADOS and the ADI-R and, for children who did not have a current cognitive assessment (within the past 12 months) confirmation of cognitive level was also completed by the psychologist on the hospital’s autism diagnostic team. If the child met inclusion criteria, parental consent, child assent, and permission to videotape treatment sessions was obtained following the approved procedures. Next, independent evaluators, trained in the administration of the assessments, conducted the pre-intervention assessments. These blinded evaluators (n = 2) were highly experienced therapists who had been licensed to practice occupational therapy for a mean of 28 years (range 26–30 years), and who had experience with working with children with ASD (mean = 19 years, range = 16–22 years). These evaluators also were trained and certified in the use of the SIPT for an average of 12.5 years (range = 9–16 years).

Following the completion of the initial assessments, the independent evaluators analyzed the assessment data (assessments are listed below) and met with the parents to identify five goals that would be addressed during the study period. These goals were scaled according to GAS Methodology (Kiresuk et al. 1994). To maintain a level of objectivity, parents did not view the goal attainment scales upon their completion or during the study period. Independent evaluators remained blind to child allocation during the study period and post-testing phases, and completed the post-intervention assessments using the same assessment battery.

### Randomization, Allocation Concealment and Implementation

Once goals were identified and scaled, children were randomly assigned using random number generations provided by the study statistician to either the treatment or UC control group using random permuted blocks within four strata^1^ based on cognitive level (hereafter referred to as IQ) and autism severity score (high IQ/high severity, low IQ/low severity, high IQ/low severity, low IQ/high severity). A high IQ was classified as a score of 85 or higher and low IQ was classified as below 84. Autism severity was determined with the ADOS using procedures to calculate severity scores described by Gotham et al. (2009), in which a lower severity score indicates less severity of autism features. A severity score of 6–10 was scored as “high severity;” a score of 4–5 as “low severity.” In total, eight children were randomized in the low IQ/high severity strata, one child randomized in the low IQ/low severity strata, 22 randomized in the high IQ/high severity strata, and one randomized in the high IQ/low severity strata. The randomization sequence and opaque envelopes with randomization allocation group (treatment or UC) were generated by the Division of Biostatistics and remained concealed until the child’s strata was determined using the criteria outlined above. Children were randomized by the second author or principal investigator in order of completion of pre-test assessment and goal scaling. The number of days between enrollment and randomization was not significantly different between the treatment group (M = 30.5, SD = 14) and UC Control group (M = 35.4, SD = 8), *t*(30) = 1.18*, p* = 0.25.

Participant children randomly allocated to the treatment group received the intervention three times per week in 1-hour sessions for 10 weeks. All parents were instructed to continue with their child’s usual weekly treatments and to document their child’s non-study related treatments on a treatment log and to report weekly if any unusual events occurred in their child’s lives (e.g. vacation, new baby). Following the study period, both groups underwent post assessment by the independent evaluators at a different location to further ensure blinding. Information on subjects’ completion of allocated intervention and attrition is displayed in Fig. 1. Participant recruitment, enrollment, randomization and retention.

### Intervention

Children in the treatment group received a manualized OT/SI intervention (Schaaf et al. 2011; Schaaf and Mailloux, in preparation) that followed the principles of sensory integration as outlined by Ayres (1972, 1979, 2005) and described in detail by Schaaf et al. (2009); Parham and Mailloux (2010); Parham et al. (2011, 2007); Mailloux and Smith Roley (2010); Schaaf et al. (2012); and Schaaf and Nightlinger (2007). The reader is referred to these sources for an in-depth description. The manualization of the intervention and examination of the treatment manual’s adherence to fidelity was conducted in a feasibility study prior to this trial, and results are described elsewhere (Schaaf et al. 2012). Importantly, following the Data Driven Decision Making Process (Schaaf et al. 2011; Schaaf and Blanche 2012) outlined in the intervention manual, assessment data were used to develop hypotheses about the sensory motor factors affecting the child’s functional behaviors and individually-tailored sensory motor activities were developed to address these factors. For example, if assessment data showed that the goal of “participate in a play activity with a peer for 10 min” may be related to poor tactile processing and praxis (hypothesis), individually- tailored sensory motor activities were designed to address tactile discrimination and improve praxis. Individually-tailored treatment activities might include activities such as using a carpeted scooter board while in the prone position to pull oneself up a ramp, then working to turn the scooter board around to ride down the ramp and land in a cushioned area of mats and pillows that are covered with various textures. In this activity, the child is experiencing total body tactile and proprioceptive sensations (from scooter board texture, actively moving muscles against resistance, and landing in textured mats and pillows) to increase body awareness and using this enhanced sensory input to plan body movements during the scooter board activity. Of note, the intervention is contextualized in play with active involvement of the child and conducted in a large gym equipped with mats, a variety of suspended swings, large balls, a climbing wall, carpeted barrels, large inner tubes and foam blocks with opportunities for active, guided, sensory motor play. The therapist facilitates the child’s ability to participate in the sensory-motor experiences in adaptive ways (e.g.: use a trapeze swing to experience proprioceptive and vestibular sensations to increase body awareness and then organize the body to hold onto the swing and jump into a large ball pit). It should be noted that this treatment is not designed to be a comprehensive treatment for autism, but rather part of a comprehensive program that includes educational, behavioral and medical services.

The intervention was delivered by three registered, licensed occupational therapists with extensive experience working with children with ASD (mean years of experience = 15, range 12–20 years), with certification in sensory integration,^2^ and who were trained on the manualized intervention. In addition, the interventionists received 3-day training by the third author and weekly consultations with the first author to discuss challenges and questions.

### Fidelity

Fidelity checks were utilized in this study to accomplish two purposes: (1) to monitor and improve provider use of the intervention manual procedures while minimizing drift in provision of services, and (2) ensure the external validity of the study procedures by documenting provider adherence to principles of intervention (Bellg et al. 2004). Treatment fidelity was confirmed using the Fidelity Measure discussed above (Parham et al. 2007). This measure has strong inter-rater reliability (0.99 for total score), with individual item inter-rater reliability ranging from 0.94 to 0.99. Validity for the measure is also strong as raters were accurately able to distinguish the manualized intervention sessions from other intervention approaches with 92 % accuracy. A score above 80/100 is considered acceptable fidelity and distinguishes this intervention from others (Parham, et al. 2007). In this study, all treatment sessions were videotaped and a random selection of 10 % (n = 51) were evaluated and rated. The mean fidelity score was 90.1 (SD = 9, Range = 53–100). Regarding the few sessions that did not reach a score of 80, additional training and consultation was provided to the therapists to support their adherence to the intervention.

## Measures

### Phenotypic Measures

#### Autism Diagnosis

Autism diagnosis was confirmed by non-study psychologists in the autism clinic at treatment site using the ADI-R (Lord et al. 1994) and the ADOS-G (Lord et al. 1999). The ADI-R is a semi-structured parent interview used to diagnose children with autism spectrum disorders and, in conjunction with the ADOS, is considered to be a gold-standard assessment for the diagnosis of ASD. The ADI-R has established validity and reliability with trained administrators (Lecavalier et al. 2006; Lord et al. 1994). The ADOS is a well-established diagnostic instrument that codes the child’s behaviors during play and interactions with the examiner. This assessment also has demonstrated validity and reliability when administered by trained professionals.

#### Cognition

Children who met criteria for an ASD diagnosis and who were interested in the study underwent cognitive testing. Measurement of cognitive level was completed using the Stanford-Binet-V (Roid 2003), the Differential Abilities Scale-II (Elliott 2007), or the Wechsler Preschool and Primary Scale of Intelligence-III (WPPSI) (Wechsler 2003).^3^

#### Sensory Assessments

Eligible participants were evaluated by independent occupational therapy evaluators to identify and describe difficulties processing and integrating sensory information using the Sensory Integration and Praxis Test (SIPT) (Ayres 1989) and the Sensory Profile (Dunn 1999).

The Sensory Integration and Praxis Tests (SIPT) are group of 17 tests that measure a child’s sensory motor abilities in the areas of tactile perception, motor planning, visual-perception, vestibular and proprioceptive processing and awareness (Ayres 1989). The SIPT is the gold standard for assessing sensory integration and praxis, and is standardized on nearly 2,000 children 4–8 years 11 months. This assessment produces standard scores for normative age groups on each of the 17 tests, was administered to all participants and findings were used to generate hypotheses about the sensory motor factors affecting the identified goals.

The Sensory Profile is a 125-item parent report of a child’s sensory behaviors using a Likert-scale format to quantify the frequency of occurrence of behaviors. The Sensory Profile is appropriate for children ages 3–10 years, and was standardized on over 1,200 children with and without disabilities. Content and construct validity has been established. Responses are summarized in six sensory processing domains of Auditory Processing, Visual Processing, Vestibular Processing, Touch Processing, Multisensory Processing, Oral Sensory Processing, five modulation areas, and three domains describing a child’s emotional and behavioral responses to sensation. The Sensory Profile was administered to all participants to characterize their sensory reactivity (i.e.: over/under responsiveness, seeking or avoidance) in the areas listed above and findings were used to generate hypotheses about sensory factors affecting identified goals.

#### Primary Outcome Measure: Goal Attainment Scaling

Goal Attainment Scaling (GAS) provides a standardized means to capture the diversity of meaningful, functional outcomes (Kiresuk and Sherman 1968). It provides a systematic process for identification of goals that are specifically relevant to individuals and their families and has been shown to be a promising outcome measure in ASD (Ruble et al. 2012). GAS has been used extensively for outcome measurement (Ruble et al. 2010; Pfeiffer et al. 2011; Miller et al. 2007; Mailloux et al. 2007) and is shown to be a valid and reliable method for measurement of progress on individualized goals for children with ASD (Ruble et al. 2012; Palisano et al. 1992). For example, Ruble et al. (2012) report good reliability when objectives are clearly measurable finding average intra class correlation between 2 study samples of 0.98 (CI 0.74–0.99) for measurability, 0.96 (CI 0.74–0.99) for equi-distance, and 0.77 (CI 0.65–0.99) for difficulty. In a study of 65 infants ages, 3–30 months, Palisano et al. (1992) found that GAS is valid as a responsive measure of motor change for infants with motor delays as “neither type or category of goals influenced the therapists’ ability to select outcomes that the infants were capable of achieving” within the 6 month intervention period (p 335). Ruble et al. (2012) concludes that GAS is a “promising ideographic approach for measuring intervention effectiveness” (p 1983). These authors recommend using a GAS template to assure goals are standardized and systematic to create reliable and valid goals, and to conduct technical checks that assess the qualities of the goal scaling to assure methodological soundness, strategies that we utilized in the current study. A technical check was completed by the second author on each GAS to assure that it met all quality markers using a technical checklist that included items based on GAS literature such as “The desired behavior/skills is observable and measurable with criteria of frequency and duration; the projected level of performance is based on the child’s current level and scaled with intervals that represent equidistance.” A mathematical method is used to calculate a T-score that represents the extent to which the goals are met (Ottenbacher and Cusick 1990) and thus, although the goals are different for each participant, the score is standardized.

**Fig. 2:**
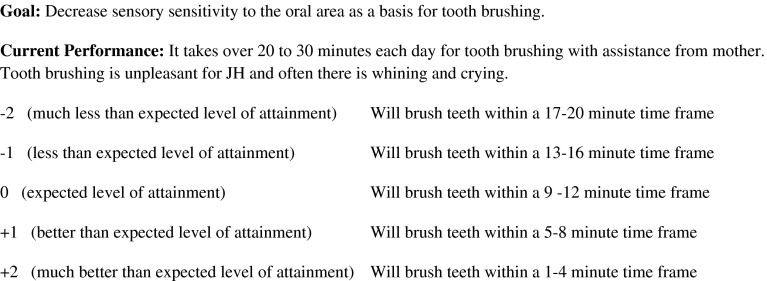
Sample goal attainment scale

Goals for each child were identified by the independent evaluators using a standard series of questions with the parent and then scaled with equally spaced probability intervals according to the procedures recommended by Kiresuk et al. (1994); Ruble et al. (2012); and Mailloux et al. (2007). To scale each goal, the independent evaluator describes the child’s current level of functioning for the specific goal and then scales it for expected level of attainment over the 10 week period (improvement) and down (regression). The probability distance between the levels of the scale is equal and equally distributed around the predicted level of performance. A score of “0” is used for expected level of attainment during the 10-week period, with scores of −1 and −2 denoting less and much less than expected level of attainment respectively; while +1 and +2 denote better and much better level of attainment than expected. Following the intervention period, the independent evaluators who were blind to group assignment conducted a standardized interview with the parents and asked parents to rate their child’s goals. A summary of the type of goals identified by parents for this study are shown in Table 2; and a sample GAS is displayed in Fig. 2.

**Table 2:** Frequency *(f)* of goal type by study group

Type of goal	OT/SI *(f)*	Usual care (*f)*
Self-care	27	25
Play	16	15
Sitting	12	9
Daily routine participation	7	1
Fine motor	5	1
Meal participation	1	0
Community participation	4	2
Communication	2	1
Self-stimulatory behaviors	3	3
Emotional regulation	3	6
Gross motor/praxis	2	3
Safety	1	2
Sleep	1	3
Impulsive behaviors	1	0
Inappropriate touching	0	4

Sample goals (italicized portion represents goal)

The child is sensitive to auditory stimuli and wakes during the night easily. Goal: Improve auditory process as a basis for* sleeping through the night without getting out of bed for 7–8 h per night*

This child hates touching food and uses a napkin to cover his food before touching it. Goal: Decrease tactile sensitivity as a basis for* eating with his fork and spoon for 50 % of the meal as appropriate*

This child has oral-sensory sensitivity and a limited food repertoire. Goal * Decrease oral sensitivity and will try 5 new foods*

This child has dyspraxia and poor tactile processing. Goal: Improve praxis and tactile processing as a basis for* putting on socks independently*

This child has tactile sensitivity and avoids contact with others. Goal: Decrease tactile sensitivity so child can tolerate* play with sibling for 5 min without supervision*

#### Secondary Outcome Measure

Pediatric Evaluation of Disability Inventory: The Pediatric Evaluation of Disability Inventory (PEDI; Haley et al. 1992) was used to evaluate a child’s self-care, mobility, and social function skills. Additionally, this assessment evaluates the amount of caregiver assistance and modification that is needed for the child to participate fully. The PEDI has been used in pediatric intervention studies, and has good psychometric properties for use as an outcome measure. Construct validity has been supported (Haley et al. 1992), and it has been used in intervention studies for children with ASD (Wong et al. 2010). Additionally, the assessment has high internal consistency, and excellent inter-rater reliability.

Pervasive Developmental Disorders Behavior Inventory (PDDBI): The PDDBI (Cohen et al. 2003) is a standardized assessment normed on children with autism or PDD between the ages of 18 months and 12 years, 5 months. This assessment evaluates the severity of a child’s autism behaviors on a number of parent-reported domains. For the purposes of this study, we chose a priori to evaluate a child’s outcomes on the domains most aligned with the targeted focus of treatment, specifically Sensory/Perceptual Approach (S/P Approach), Ritualisms/Resistance to Change (R/R), and Arousal Regulation Problems (Arouse) domains. The PDDBI has strong parent-reported test–retest validity over a 6-month interval (S/P Approach *r* = 0.63, R/R *r* = 0.82, Arouse *r* = 0.82), and has demonstrated construct, criterion and concurrent validity.

Vineland Adaptive Behavior Scales-II (Parent Report): The Vineland Adaptive Behavior Scales II (VABS-II) (Sparrow et al. 2005) was used to assess adaptive behaviors needed for participation in home, school and community activities at pre and post assessment. The VABS-II is a standardized, norm-referenced measure that evaluates adaptive behavior in four domains: Communication Skills (Receptive, Expressive, Written), Daily Living Skills (Personal, Domestic, Community), Socialization Skills (Interpersonal Relationships, Play and Leisure, Coping), and Motor Skills (Gross, Fine). It has good subdomain reliability with approximately 75 % of subdomain scores having a value of 0.75 or greater. Inter-rater reliability is considered good for a sample aged 7–18 years, and ranges from 0.81 to 0.71 for domain and subdomain scores, and is even higher for younger children (0.83). Internal consistency is considered good at 0.80 and test-re-test reliability is high, exceeding 0.85 (Sparrow et al. 2005).

#### Sample Size

The study was designed to gather preliminary efficacy information about the intervention with respect to the primary outcome of GAS. For this primary outcome measure we calculated power to detect different effect sizes for a given sample size. With 32 subjects we have 78 % power to detect an effect size of 1, and greater than 80 % power to detect effect sizes greater than 1 using a two-sample *t* test with a two-sided type-I error rate of 5 %.

## Results

Our main goal was to evaluate the effects of the intervention on parent-reported, individual goal attainment using GAS (primary outcome). Secondarily, we evaluated the effects of the intervention on sensory behaviors, functional and adaptive behaviors using the PDDBI, PEDI, and the VABS II. Normality of primary and secondary outcome measures was evaluated prior to evaluating group differences. Scores on these secondary outcomes were not normally distributed and thus, non-parametric statistical tests were used to account for violation of the assumption of normality. In addition, although differences in baseline scores were not significantly different between the groups, on some outcomes differences within groups at baseline would be considered clinically meaningful. To account for variability in baseline scores, we used change scores in the analyses. Given the range of pre-treatment scores, within-person change was viewed as the most clinically relevant post-treatment score. An alpha of 0.05 was used for all comparisons. Data reported represents numbers of subjects with complete data sets (see reasons for attrition in Fig. 1).

### Evaluation of Treatment Effects on Goal Attainment

To test the main effect of the treatment, we conducted a two-tailed independent samples *t*-test to evaluate the difference in goal attainment between the groups. Results revealed a significant difference between the treatment (M = 56.53, SD = 12.38, n = 17) and UC (M = 42.71, SD = 11.21, n = 14) groups on the GAS with the treatment group achieving significantly higher scores (*t*(23) = −3.23, *p* = 0.003, ES = 1.2).

### Evaluation of Treatment Effects on Functional Behaviors

To test the effect of the treatment on functional behaviors we compared the change from baseline to end of treatment for each of the PEDI subscales using the Wilcoxon Rank Sum test. We used median change scores to control for non-normal distributions. Results reveal significantly greater change (improvement) for the treatment group in comparison to the UC control group on Self-Care Caregiver Assistance subtest (*p* = 0.008) and Social Function Caregiver Assistance (*p* = 0.039). Of note, the treatment group also showed greater improvement on the Social Functions subtest (*p* = 0.097) and the Self-Care Functional Skills subtest (*p* = 0.198). The findings from the PEDI are displayed in Table 3. In addition to reporting median change scores, we also report mean and standard deviations for each subscale as these were used to calculate effect sizes.

**Table 3:** Group differences on change in standard scores on pediatric evaluation of disabilities inventory

	Control			Experimental			Significance	Effect Size^a^
	Median	Mean	SD	Median	Mean	SD		
Functional skills^b^								
Self-care	1.7	1.12	5.6	3.7	10.2	22.6	0.198	0.5
Mobility	0	6.38	15.1	0	6.57	23.8	0.69	0.0
Social	1.1	4.4	13.8	4	9.3	17.4	0.097	0.3
Caregiver assistance								
Self-care	1.3	−0.43	8.6	12.2	16.6	23	0.008**	0.9
Mobility	0	0.22	11.8	0	4.8	24.1	0.68	0.2
Social	0	−1.8	19	13.5	14.4	23.4	0.039*	0.7

^a^Mean and standard deviations are based on raw scores. Effect size is presented using the difference in means divided by the pooled standard deviation

^b^Functional Skills represent actual skills completed by child whereas caregiver assistance represents the amount of assistance that the caregiver provides

** *p* < 0.001; * *p* < 0.05

### Evaluation of Treatment Effects on Autism Behaviors

To test the effects of the treatment on sensory/perceptual approach behaviors, arousal regulation and ritualism/resistance to change, we compared the change from baseline to the end of treatment on these PDDBI subscales using the Wilcoxon Rank Sum test. Given that lower scores indicate a decrease in autism behaviors, a greater negative change scores indicates a better response. As shown in Table 3, there were no significant differences in autism behaviors at post-treatment between the groups, although changes for the treatment group approached significance in the Sensory Perceptual Behaviors Subscale (*p* = 0.064) (indicating a decrease in autism behaviors) and were also lower in the Arousal Regulation subscale (0.38).

### Evaluation of Treatment Effects on Adaptive Behaviors

To test the effects of the treatment on adaptive behaviors we compared the change in standard scores from baseline to the end of treatment each of the Vineland-II subscales and the Adaptive Behavior Composite Score using the Wilcoxon Rank Sum test. As shown in Table 3 there were no significant differences in adaptive behaviors, although the treatment group improved more than the UC Controls in all subscales.

## Discussion

Interventions to address difficulty processing and integrating sensory information are frequently used as part of a comprehensive approach for individuals with ASD. However, the evidence is compromised by methodological limitations in existing studies. Thus, there is a need for more evidence with a well-characterized sample using a manualized protocol following the principles of sensory integration and measurement of fidelity. The current study is one of the first randomized trials to meet this level of rigor (Table 4).

**Table 4:** Group differences on change scores of pervasive developmental disorders behavioral inventory

	Control			Experimental			Significance	Effect size^a^
	Median	Mean	SD	Median	Mean	SD		
S/P Approach	−0.05	−0.67	5.9	−5	−5.9	10.8	0.06	−0.6
R/R	−2	−1.77	6.3	−2	−6.5	13.7	0.57	−0.4
Arouse	−3	−3.3	6.0	−6	−7.1	11.6	0.38	−0.4

*S/P Approach* sensory/perceptual approach, *R/R* ritualisms/resistance to change, *Arouse* arousal regulation problems

^a^Effect size is presented using the difference in means divided by the pooled standard deviation

Our main finding is that subjects with ASD who were randomized to treatment scored significantly higher on our primary outcome measure, GAS, than those who received UC. Secondarily, we found that the children in the treatment group scored as needing significantly less caregiver assistance during self-care and social activities and showed a trend toward higher skills in these areas. Further, sensory behaviors in the treatment group decreased more than in the UC group and this difference approached significance (Table 5).

**Table 5:** Group differences on change in in standard scores on vineland adaptive behavior scales—II

	Control			Experimental			Significance	Effect size^a^
	Median	Mean	SD	Median	Mean	SD		
Communication	1	−3.38	18.6	1	5.06	10.9	0.20	0.6
Daily living								
Skills	0	−3.0	18.5	4	4.2	11.6	0.18	0.5
Socialization	−2	−6.7	21.8	3	3.8	11.8	0.29	0.6
Composite	0	0.0	8.1	2	15.1	44.7	0.30	0.4

^a^Mean and standard deviations are based on raw scores. Effect size is presented using the difference in means divided by the pooled standard deviation

The primary outcome for this study was the score obtained on GAS and we found that the children receiving the treatment scored significantly higher (*p* = 0.003) than the controls on goal attainment with an effect size of 1.2. Our finding is consistent with Pfeiffer et al. (2011) who found that children with ASD who participated in 6-week program of occupational therapy using sensory integration made significantly greater gains in their individualized goal attainment scale scores in comparison to those who received a fine motor intervention. Goal attainment scaling is a method to individualize and quantify goals for clinical populations. It has been used extensively in the clinical literature, and is recommended as an outcome measure in randomized control trials of psychosocial interventions in ASD (Ruble et al. 2012) such as the current study. Of note, the procedures we utilized in constructing scaled goals were consistent with the recently published recommendations of for its use (Ruble et al. 2012) including that: benchmarks were carefully constructed, goals were scaled at equal intervals, and rating of goals post intervention was based on parent interview by an independent evaluator blind to study condition. Although adherence to these conditions increases the reliability and validity of GAS (Ruble, et al. 2012) our findings must be interpreted with caution given that the parents were not blind to the intervention.

Two valuable aspects of GAS are that it provided a means to individualize goals based on each child’s individual needs *and* to identify areas that are important to the parents. Individualization is an important aspect of treatment given the heterogeneity and developmental nature of ASD as it is likely that each child has a unique set of pre-treatment characteristics that impact the choice of goals and outcomes (Stahmer et al. 2011). Further, utilization of goals that are important and meaningful to the parents assures that the primary stakeholders (families of children with ASD) needs are being addressed. This is an important aspect of any intervention and is in keeping with contemporary trends in intervention research (PCORI 2013; Melnyk and Morrison-Beedy 2012). For the current study, individual goals were based on parent-identified areas of need and assessment data that were established prior to treatment allocation. Many of the goals for the treatment and UC controls were similar in type as shown in Table 2. The most frequent goals were related to self-care including goals such as greater independence in dressing, feeding, toileting or grooming activities (treatment = 27, UC = 25). The second and third most frequent goals were about play (treatment = 16, UC = 15); and sitting for participation in activities such as synagogue or dinner (OT/SI = 12, UC = 9). There were some minor differences in goal type between the groups; the treatment group had more goals related to fine motor skills (OT/SI = 5, UC = 1) and participation in daily routines (treatment = 7, UC = 1); whereas the usual care group had more goals related to emotional regulation (treatment = 3, UC = 6), sleep (treatment = 1, UC = 3) and inappropriate touching (treatment = 0, UC = 4).

A second aspect of individualization that is important for ASD research and practice is that intervention strategies were tailored to each child’s assessed areas of need. In this study, the Data Driven Decision Making Process (Schaaf, in press; Schaaf et al. 2012; Schaaf and Benevides 2011; Schaaf and Blanche 2012) was used to individually tailor treatment activities to address the specific sensory-motor factors that were hypothesized to be affecting each participant’s goal attainment and functional skills. The treatment utilizes individually tailored sensory motor activities at the just right challenge with a playful approach to facilitate the child’s adaptation to promote function. Thus, the focus of treatment is on each individual’s sensory motor factors hypothesized to be impacting function, but importantly, the expected outcomes are functional behaviors. It is likely that this individualization was an important aspect of the positive findings of this study, and should be modeled in future studies.

In terms of functional behaviors, the children in the treatment group significantly decreased their need for caregiver assistance on self-care and social activities in comparison to the UC controls on the PEDI. In addition, they also showed a trend toward improvement in self-care and social skills. Thus, not only did the caregivers rate the children in the treatment group as needing less assistance from them in these activities, they also rated their skill level higher. These secondary outcome data should be interpreted with caution given that we completed multiple comparisons, however, these findings are consistent with the philosophy of the treatment approach—that adequate processing and integration of sensory information provides an important foundation for participation in functional, meaningful activities (Ayres 2005). Of note, the PEDI has been shown to have good reliability and validity as an outcome measure of functional behaviors (Nichols and Case-Smith 1996).

In terms of the sensory-motor factors that may underlie these findings, in this cohort many participants in both groups showed deficits in sensory modulation and praxis (measured via the Sensory Profile and the SIPT), and thus, the intervention was tailored to address these areas. Improvements in sensory modulation and praxis skills therefore, may underlie the gains seen in self-care and social skills. In regard to sensory modulation (over or under-reactivity to typical levels of sensation), the individually-tailored treatment for these children included a focus on activities that facilitated sensory modulation and regulation of behavioral responses to these sensory experiences. As the child’s ability to modulate sensation improved, it is likely that their behavioral regulation also improved and subsequently they were better able to participate in self-care and social activities. Interestingly, the subjects in the treatment group did show a decreasing trend of negative sensory behaviors on the Sensory Perceptual Behaviors Subscale of the PDDBI and this approached significance (*p* = 0.064), supporting this interpretation.

Similarly, it is possible that the intervention also had an impact on praxis. Praxis involves the ability to conceive of, plan, and organize goal-directed motor actions (Ayres 1989; Dziuk et al. 2007) and is related to adequate processing and integration of body sensory information (tactile, vestibular and proprioception). The intervention aimed to facilitate body awareness and praxis through individually-tailored, active, sensory-motor activities rich in tactile, proprioceptive and vestibular sensations. Many self-care activities such as dressing require adequate body awareness and thoughtful planning and execution of motor skills. Thus, it is possible that improved body awareness and praxis had a positive impact on ability to carry out these self-care tasks. Similarly, social interactions require constant processing of varied, often unpredictable sensations and the need for spontaneous responses (i.e.: praxis) and are likely affected by difficulty processing and integrating sensory information related to the body (Hilton et al. 2007, 2010; Baker et al. 2008; Ashburner et al. 2008; Reynolds et al. 2011; Watson et al. 2011; Hochhauser and Engel-Yeger 2010). Thus, as the children’s praxis improved, it is plausible that their ability to adaptively plan and carry out social interaction activities also improved and they became more independent. Further testing of these potential relationship is needed and in order to validate the idea that improvements in sensory modulation and praxis were related to improvements in functional skills, it will be important in future studies to specifically measure any changes in in these factors and their relationship to changes in functional skills such as self-care and socialization. In this study we were limited by the lack of instruments validated to measure change in these factors for this population within our 10-week intervention period. The SIPT is not recommended as a pre-post-test measure for periods shorter than 8–12 months (Ayres 1989) and its utility for shorter intervention periods has not been tested. Similarly, the Sensory Profile has not been validated for use as a pre-post assessment (Dunn 1999) although there is emerging data that test–retest reliability of certain sub-scores may be utilized in this way. Until these measures are validated for use as outcome measures in shorter intervention periods, or outcome measure to evaluate change in sensory functions are validated, future studies should consider a longer intervention period so that these assessments can be used to measure change in sensory-motor skills and determine their relationship to any changes in functional skills and adaptive behavior.

In terms of the proposed mechanism underlying the positive findings in this study, one explanation is that the intervention impacted neuroplasticity—the ability of the nervous system to be shaped and influenced by experience. It is well regarded in the neuro-developmental literature that early sensory motor experiences promote neuroplasticity and enhance the capacity of the brain to adapt to environmental challenges (Shonkoff and Phillips 2000; Ayres 1972; Dawson et al. 2012). Thus, it is possible that through the process of neuroplasticity that the children became more independent in their functional skills as their ability to process and integrate sensory information improved. Further testing of this assumption is needed using methods that evaluate nervous system activity pre and post intervention. There is some preliminary evidence that change in neural activities results from enriched environments. For example, Dawson et al. (2012) showed that more organized EEG activity occurred in children with ASD who also made gains in the Denver Early Start Program; and Miller et al. (2007) showed that electrodermal activity, a measure of sympathetic nervous system activity, showed a trend to decrease (expected direction) following a sensory-enriched intervention in subjects who were previously sensory hyper-reactive. An important next step in this research will be to measure changes in brain activity that may be concurrent with improvements in adaptive behaviors and individual goals as suggested by Schaaf et al. (2013).

In summary, our data provide preliminary support for the efficacy of a manualized intervention designed to address difficulties processing and integrating sensory information for children with ASD. We show improvements in our primary outcome—Goal Attainment as well as our secondary outcome measures showing improvements in self-care and social activities reflected by decreased caregiver assistance. These findings should be interpreted cautiously until they are replicated in a larger sample size. In addition, in future studies it would be useful to include additional outcome measures that rely on direct observation of goal attainment and sensory behaviors to provide further validation of GAS findings. It will be important to supplement parent reported data with direct observational measures. It will also be important to include a longer intervention period in future studies and follow-up testing to determine if the observed changes are maintained. Finally, although we randomized subjects based on autism severity and cognition, we were not able to include these strata in our analysis due to our sample size. Future studies would be strengthened by the inclusion of a larger sample so that impact of potentially confounding variables on treatment outcomes can be evaluated. Of note, almost all of our participants (30 of 32 children, or 94 %) demonstrated high severity of autism, and 22 or 68.75 % also had high IQ. It would be useful if future studies utilizing this intervention include children with low severity and/or low cognition to determine if the findings from this study are replicated with this sample. Similarly, our sample of convenience resulted in a sample with little ethnic diversity and future studies should make an effort to include participants from more diverse backgrounds. Despite these limitations, this study provides evidence that this intervention may be a useful adjunct to a comprehensive intervention program for individuals with ASD who have functional and behavioral challenges related to difficulty processing and integrating of sensory information.

## Footnotes

1 Although we randomized based on these strata, we did not complete subgroup analyses based on strata due to small sample size.

2 Certification in sensory integration requires participation in 120 h of post-graduate course work offered and 40 hours of practice under the guidance of an experienced clinician trained in sensory integration (Parham et al. 2007).

3 One child was tested with the Woodcock-Johnson Test of Achievement.
